# Postshock Pacing in Cardiac Arrest: A Concise Review

**DOI:** 10.1155/emmi/9067144

**Published:** 2025-09-05

**Authors:** Wojciech Telec, Salwan Al-Saad, Lukasz Karbowski, Tomasz Kłosiewicz, Artur Baszko

**Affiliations:** ^1^2nd Department of Cardiology, Poznan University of Medical Sciences, Poznan, Poland; ^2^Cardiology Research Group, English Students' Research Association, Poznan University of Medical Sciences, Poznan, Poland; ^3^Department of Medical Rescue, Poznan University of Medical Sciences, Poznan, Poland

**Keywords:** advanced life support, cardiac arrest, cardiopulmonary resuscitation, postshock pacing

## Abstract

Following an administered shock in cardiac arrest, the heart commonly experiences a short phase of inability to efficiently perfuse. Despite being a commonly used feature in the ICD population, postshock pacing (PSP) is yet to be adequately explored for its utility in this pulseless phase. Notably, an overwhelming proportion of available data for transcutaneous pacing in spontaneous cardiac arrest stem from the 1980s and 1990s and revolve largely around nonshockable, as opposed to shockable rhythms. The lack of large-scale clinical trials assessing the efficacy of transcutaneous PSP and the considerable advancements in technology and training facilities since the 1990s indicates a need for reevaluation of current understanding of PSP and its applicability in cardiac arrest. Shedding light into the possible implications of transcutaneous PSP in emergency setting cardiac arrest may not only reshape the current protocols of ALS but also carry the potential of improving survival rates. This concise review serves as a summary of the existing knowledge on the subject of PSP and reveals further possible directions for the development of this therapy.

## 1. Introduction

Advanced life support (ALS) is a well-known fundamental protocol in cardiovascular emergencies. Emphasis on the return of normal body perfusion with minimal delay and urgency to sustain vital organs is at the core of ALS [1]. Continuous efforts to fine-tune already existing guidelines and explore superior maneuvers are of utmost importance, with potentially life-saving impact.

During routine ALS, postshock pulseless electrical activity remains a pressing issue that must be attended to. A significant proportion of patients remain in a transient nonperfusing phase following defibrillation, and only rarely is a return of pulse observed immediately following the shock [2–4]. Such a stagnant phase of absence of palpable pulse can last for more than 2 min and, if left unattended, can have detrimental consequences on survival [2]. In cases where some electrical activity is restored following defibrillation, profound postshock bradycardia and even atrioventricular blocks (especially in those with administered antiarrhythmic drugs) have been noted [3, 4]. As such, current ALS guidelines explicitly state the need to continue chest compressions following the successfully administered defibrillation [1, 5]. Such a protocol maintains at least a partial perfusion until the return of sufficient heart contractility or until the next shock. Externally pacing the heart during this vulnerable nonperfusing phase is currently not indicated by ALS guidelines. On the other hand, in the population of patients with implantable cardioverter-defibrillator (ICD), postshock pacing (PSP) is a feature heavily utilized. PSP has shown potential benefit for ICD patients experiencing cardiac arrhythmias, by pacing and supporting cardiac muscle contraction following a shock [6]. However, this feature has not yet been heavily scrutinized, especially for its use in spontaneous adult ALS situations.

Postshock transcutaneous pacing potential benefits, alongside routine ALS and chest compression protocol, still remain largely unexplored. A concise review of existing literature may elaborate on the current understanding of transcutaneous PSP in cardiac arrest and may further highlight possible directions for the development of this therapy.

## 2. Transcutaneous Pacing in Cardiac Arrest

In the mid-1950s, Zoll and colleagues pioneered the use of external transcutaneous electrical stimulation in cases of cardiac arrest [7]. They described the use of the technique in 8 cardiac arrest patients (of various etiologies) and reported promising outcomes. A period of limited data followed as transvenous pacing emerged [8], which circumvented some primary complications the original transcutaneous devices carry (such as severe pain and damage to underlying muscle tissue). Nonetheless, gradual refining of techniques and tools reestablished transcutaneous pacing as the first-line method.

The 1980s saw a large increase of reports assessing the efficacy of transcutaneous pacing in various cardiac complications. As one of the earliest reports, Falk et al. affirmed the practical use of out-of-hospital transcutaneous pacing in cardiac arrest [9]. In their 19 cases, they reported some promising results in a bradycardia patient (1 patient); however, they stated poor prognosis despite electrical stimulation in those with asystole (18 patients). Similarly, Dalsey and colleagues utilized transcutaneous pacing in asystole and bradycardia patients (with no pulse or severe hypotension), in 30 and 22 emergency department (ED) patients, respectively [10]. While 4 had a return of blood pressure, no patient survived till discharge from hospital. A wave of studies echoed similar findings, stating no improved results in survivability of paced cardiac arrest patients (mainly asystolic patients), both in and out of the hospital settings [11–15]. In their short review and report of 12 out-of-hospital cardiac patients, who received transcutaneous pacing, Olson and colleagues placed large emphasis on the requirement for shorter delays in pacing initiation [16]. Syverud et al. reaffirmed the importance of timing by comparing outcomes in cardiac arrest patients who were paced within 5 min of the arrest (5 patients) to those who received it between 5 and 20 min postarrest (14 patients) [17]. Two patients with shorter delay in pacing had a return of full neurological and cardiac function, while the latter group was significantly less likely to develop a pulse or have a full return of neurological function. However, no statistical difference was noted in the overall clinical outcome between the 2 groups—perhaps attributed to the limited sample size.

A few larger studies with bigger sample sizes also reported coherence in results [18, 19]. In their controlled clinical trial, Barthell and colleagues were able to further exemplify the role of transcutaneous pacing by comparing 103 paced cardiac patients to 136 nonpaced [20]. Their sample consisted largely of asystolic or electromechanical dissociation patients (pulseless, HR < 70 BPM) and some severe bradycardia patients. The study concluded that prehospital transcutaneous pacing made no significant difference in outcome of electromechanical dissociation or asystole patients, but did show positive results in bradycardia patients. Furthermore, in a decisive study in the 1993 that spanned over 3 years, Cummins and colleagues directed their emergency staff to initiate cardiac pacing, as early as possible, in a portion of the primary asystolic or post-defibrillation asystole cases [21]. Their results show that the paced group (112 out of 278 cases) had no significant improvement (or deterioration) in survival or hospital admission rate. They concluded that incorporating transcutaneous pacing into standard protocol for out-of-hospital asystole cases would be of no benefit. A summary of the studies assessing transcutaneous pacing in cases of cardiac arrest, and types of rhythms paced, can be observed in Table 1.

As is clearly demonstrated by the overwhelming majority of studies, transcutaneous pacing in cardiac arrest scenarios has been considerably scrutinized for its applicability in asystolic cases.

## 3. Transcutaneous Pacing in Bradycardia

In contrast to its poor utility in complete absence of electrical activity, transcutaneous pacing has shown some favorable outcomes in profound bradycardia, bradyarrhythmias, and other arrhythmic patterns. It must be noted that some important factors, a crucial one being time delays in initiation of pacing, vary between studies and have therefore led to few studies reporting conflicting data [19]. Nonetheless, in the case of symptomatic bradycardia, early studies showed large consistency in positive results from the use of transcutaneous pacing, especially if the patient was unresponsive to pharmacological treatment [9, 22–25]. In 1999, the American Heart Association (AHA) recommended the setup of transcutaneous pacing patches in myocardial infarction (MI) patients with bradycardia (< 50 bpm) and notable signs for hypotension (< 80 mm Hg, systolic pressure) [26]. Such a hemodynamic scenario was stated as a class 1a indication for transcutaneous pacing in MI patients. More recent data have put into question the true efficacy of transcutaneous pacing in bradyasystolic cardiac arrest [27]. In a 2006 systematic review, Sherbino et al. highlighted the inadequacy of conclusive data to support the use of transcutaneous pacing in prehospital symptomatic bradycardia [28]. In the 2018 AHA guidelines, transcutaneous pacing was considered a temporary and viable, yet less strongly recommended, option for symptomatic bradycardia [29]. Despite the need for large controlled trials, current literature is leaning toward a favorable use of transcutaneous pacing in cardiac arrest cases of low heart rate, especially if accompanied with symptoms resistant to pharmaceutical treatment [30].

It is evident that the medical literature has revolved largely around pacing in nonshockable rhythms, namely, asystole. Its use for symptomatic bradyarrhythmias in MI has also been highlighted. The utility of pacing following defibrillation in cardiac arrest (PSP) and in shockable rhythms is an important aspect that should also be safely studied. Unfortunately, extremely limited human data are available concerning the efficacy of transcutaneous pacing in such scenarios. In animal models, synchronizing the cardiac tissue via pacing immediately after the shock led to some successful defibrillation attempts and cases of complete termination of ventricular fibrillation (VF) [31, 32]. In a 2010 case report, Nikolic et al. reported successful resuscitation, due to transcutaneous pacing, after five failed defibrillation attempts in an asystolic cardiac arrest patient [33]. Despite the late implementation of pacing, the patient benefitted from PSP and survived till discharge. Since shockable and nonshockable rhythms are distinctly different physiological phenomena, results from cardiac arrest studies utilizing pacing should be scrutinized for which rhythms are being paced and during what phase of cardiac arrest. Given the large focus on asystolic cases (nonshockable rhythms) in previous studies, the efficacy of postshock transcutaneous pacing in shockable rhythms remains unclear.

There is substantial evidence that significant bradycardia and AV blocks may occur following an administered electrical shock in cardiac arrest [2–4]. This is compounded by the fact that some administered drugs during ALS, such as amiodarone, can cause hypotension, bradycardia, or conduction blocks [34]. In most cases, the heart is unable to create an efficient pulse soon after the electrical stimulation [1, 3, 4, 35, 36], and guidelines have therefore explicitly stated the need for minimally delayed postshock chest compressions [1, 5]. Transcutaneous pacing is an outstanding candidate yet to be analyzed as a solution for this postshock phenomenon. Having shown some promising results in symptomatic bradycardia, and with antiarrhythmic features, PSP carries undeniable potential to prove beneficial in shockable cardiac arrest cases.

## 4. PSP in Implantable Devices

PSP is an already well-established feature in patients with ICD and cardiac resynchronization therapy with defibrillation (CRT-D). During implantation, the ICD or CRT-D can be programmed so that following defibrillation, the device initiates pacing at a given rate that is usually higher than default back-up ventricular pacing; in most cases, PSP is programmed at 70 bpm for a duration of 10–30 s following shock [37, 38]. Although this feature is commonly utilized, its true efficacy is yet to be extensively studied. In an abstract published by Verma and colleagues, they assessed PSP in 314 implanted ICDs (728 VF/ventricular tachycardia [VT] episodes) [39]. They estimated that PSP was initiated efficiently and with success in almost 1 in 4 induced VF/VT episodes. PSP captured the ventricles in all episodes, and no cases of induced arrhythmias due to pacing occurred. However, in their investigation, the shock was delivered in a nonspontaneous environment (patient was under general anesthesia or sedation). In another study, it was estimated that roughly half the ICD patients who experienced ventricular arrhythmias in spontaneous situations required pacing and were successfully paced following shock without any adverse reactions to PSP such as proarrhythmia or undersensing [6]. The authors concluded that PSP's successful applicability was influenced by factors such as age, history of ischemic cardiomyopathy, chronic obstructive pulmonary disease, and necessity for permanent pacing prior to the shock. Budeus and colleagues measured and compared blood natriuretic peptide (BNP) levels in 780 consecutive patients that underwent defibrillation threshold test (DFT) after implantation of ICD or CRT-D. They analyzed BNP levels prior to and after the shock in subgroups of patients without PSP and with PSP programmed with different pacing frequencies and duration. The most favorable trend in BNP levels (the lowest increase in BNP) was achieved with PSP frequency of 90 bpm and duration of 60 s. They concluded that clinical outcome might be improved by optimized PSP as BNP levels are widely known to correlate with cardiac stress and long-term outcome [40, 41]. Subcutaneous ICDs (S-ICDs), the devices normally lacking the function of pacing, have the capability to deliver pacing output following the shock. Abbott and colleagues described the case of unsuccessful PSP in a young patient with Ebstein's anomaly and implanted earlier permanent pacemaker (PPM) and more recently S-ICD. Transvenous PPM was unable to capture the heart rhythm following S-ICD shocks during defibrillation threshold testing. Moreover, complex device to device interactions led to undersensing of ventricular arrhythmia by S-ICD and failure to deliver the shock. Patient required removal of both systems and implantation of transvenous ICD [42].

All of abovementioned studies emphasize both the importance of PSP and the need for further studies in order to elucidate the critical role of this modality in cardiovascular emergencies. Most of our knowledge of PSP is based on observations in the electrophysiology laboratory, and it may not reflect the pathology associated with spontaneous out-of-hospital cardiac arrest. To conclude, despite the lack of thorough studies, PSP is generally believed to carry more benefit than harm and therefore continues to be used in the vast majority of ICD/CRT-D population.

## 5. PSP in Patients With Wearable Cardioverter Defibrillator (WCD)

Patients who are at high risk of SCD but are currently not eligible for an ICD might benefit from WCD use [43]. These devices do not have the capability of pacing; they detect the arrhythmia and provide defibrillation shock through external electrodes placed on the chest. Berger et al. report in the study based on 313 cases of out-of-hospital deaths while wearing the WCD that postshock asystole and postshock bradycardias are very common (65.2% and 35.5%, respectively). Of 8 patients with PPMs, 1 device became nonfunctional and 7 exhibited noncapture after the shocks. This study gives insight into the terminal rhythms of patients who die after shocks delivered by WCD and underline the high occurrence of asystole and bradycardias following the shock. Importantly, the study reports unsuccessful PSP in all 8 patients with implanted pacemakers [44]. Summary of studies assessing PSP is presented in Table 2.

## 6. Possible Complications

Transcutaneous pacing, like any medical intervention, carries the risk for possible complications that must also be discussed. Despite having largely improved since being first introduced, skin burns and pain should still be considered. Regular assessment of contacted skin and electrode repositioning is helpful if prolonged pacing is required. Another noncardiac-related complication includes hiccups or coughing due to diaphragm stimulation [45]. These complications carry perhaps the least severe threat to the patient and may be avoided to some extent with simple maneuvering or administration of analgesic. Cardiac complications are the most feared, especially due to the fragility of the heart during a cardiac arrest. There is a risk of exacerbation of tachyarrhythmias into more complicated and resistant arrhythmias. However, in PSP patients with ICD, such a complication has not been commonly reported [6, 38]. Conversion to bradyasystolic rhythm has been reported in some cases of VF pacing [13, 23]. If PSP is to be considered for future studies, careful observation into development of complications is required.

## 7. Technical Considerations

In order to safely perform studies assessing the efficacy of transcutaneous PSP in shockable rhythms, few technical aspects must also be addressed. Directing efforts to attain ICD records from patients with and without PSP configuration may be a first observational step in the right direction. Approval from ethical committees may even allow for crucial data from deceased patients. Furthermore, since most external pacers are accessible and incorporated into modern emergency equipment [45], optimizing protocol and staff training become the core hurdle. Formulating a protocol that incorporates pacing without significantly jeopardizing current ALS maneuvers, in shockable rhythms, is key for large randomized control trials. Take, for instance, Morrison et al., who successfully illustrated the feasibility of conducting randomized control trials for out-of-hospital transcutaneous pacing in patients with unstable bradycardia [46]. More studies of similar nature may help in assessing the safety and clinical implications of utilizing PSP in cardiac arrest.

The survival rate of SCA is primarily influenced by the quality of delivered resuscitation. This is a factor that is highly dependent on the persons providing care. Factors such as algorithm compliance, team cooperation, and nontechnical skills have a proven impact on survival rates. Furthermore, the introduction of an additional procedure into the ALS algorithm must not interfere with the core intervention, which is high-quality chest compression.

Currently, emergency staff are directed to continue chest compressions immediately after administering the shock [1, 5]. Studying the possibility of including a short, yet effective, window of pacing prior to resuming compressions is fundamental in performing safe, large-scale studies. Assessing the time factor and applicability of any suggested PSP protocols in simulation centers allows for an unprecedented level of preparation prior to moving into clinical studies.

In a simulation study conducted on a group of paramedics, it has been shown that the use of PSP as an additional procedure does not affect the quality of compressions [47]. Moreover, the implementation of 30 s course of PSP had no negative impact on adherence to ALS protocol [48]. Simulation studies have well-known limitations. However, this method may be helpful in applying newly developed procedures into clinical environment [49]. The results of the abovementioned studies raise the hope that the use of PSP can be applied in clinical trials without affecting the quality of standardized activities.

## 8. Practical Considerations

**Figure 1:**
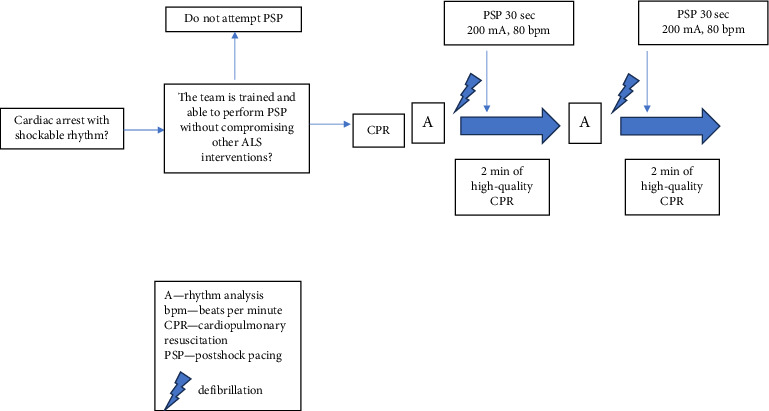
A proposal for positioning postshock pacing into the advanced life support algorithm.

Current guidelines on resuscitation do not recommend routine PSP, and the authors of this review strongly recommend to refer to the latest guidelines from authoritative sources like the AHA or European Resuscitation Council (ERC) for the most current protocols of ALS cases [1, 5]. However, PSP should not be excluded as a treatment option and some studies mentioned above support the hypothesis that there might be cases in which patients will benefit from pacing the heart immediately after the shock. Simulations have shown that trained and experienced team is able to deliver PSP without compromising resuscitation quality and adherence to the ALS protocol [43]; therefore, based on these simulations, the authors present the decision-making flowchart that might prove helpful to prepare the caregiver team for performing modified ALS algorithm with PSP (Figure 1). We strongly recommend to practice PSP scenarios first in a simulated setting and consider this therapy only when all members of the team are well trained and able to perform this task without compromising any other significant interventions (resuscitation quality, pharmacotherapy, imaging, etc.).

## 9. Summary

Following an administered shock in cardiac arrest, the heart commonly experiences a short phase of inability to efficiently perfuse. Current guidelines direct emergency staff to resume chest compressions immediately after defibrillation to avoid any consequential effect on organ perfusion. Despite being a commonly used feature in the ICD population, PSP is yet to be adequately explored for its utility in this pulseless phase. Notably, an overwhelming proportion of available data for transcutaneous pacing in spontaneous cardiac arrest stem from the 1980s and 1990s and revolve largely around nonshockable, as opposed to shockable rhythms. The lack of large-scale clinical trials assessing the efficacy of transcutaneous PSP and the considerable advancements in technology and training facilities since the 1990s indicates a need for reevaluation of current understanding of PSP and its applicability in cardiac arrest. The unparalleled level of preparation, offered by modern-day facilities, may allow for a safer transition into vital clinical studies. Shedding light into the possible implications of transcutaneous PSP in emergency setting cardiac arrest may not only reshape the current protocols of ALS but also carry the potential of improving survival rates.

## Figures and Tables

**Table 1:** Summary of studies assessing transcutaneous pacing in cardiac arrest.

Study	Country, year	Number of paced patients	Types of rhythms paced (# of cases)	Study conclusions
Tancredi et al. [8]	USA, 1956	8	Nonshockable rhythms: Asystole (8)	Proposed external electric stimulation as a potential treatment for asystole in cardiac arrest

Dalsey et al. [10]	USA, 1983	19	Nonshockable rhythms: Asystole (15) Idioventricular rhythm (4)	Highlighted technical feasibility of external pacing in out-of-hospital setting but noted no improvements in outcome of paced patients. Seven patients developed electrocardiographic evidence of pacemaker capture, although only 2 had palpable pulses

Clinton et al. [11]	USA, 1985	52	Nonshockable rhythms: Asystole (30) Pulseless/bradycardia (22)	26 patients (50%) had successful ECG capture; 8 of them (31%) developed a pulse. No patient survived till discharge. All patients paced efficiently transvenously were also successfully paced transcutaneously

Knowlton and Falk [12]	USA, 1985	37	Nonshockable rhythms: Asystole (16) Complete heart block (4) Sinus bradycardia (2) Nodal bradycardia (1) AF with bradycardia (2) Electromechanical dissociation (1) Idioventricular rhythm (10) Torsades de pointes (1)	Of 8 patients who had responded to pacing, 6 survived. Of 28 nonresponders, 5 survived (*p* < 0.005). External pacing applied to significant bradycardia showed effective improvement; however, cases of asystole did not benefit from transcutaneous pacing

Eitel et al. [13]	USA, 1986	26	Nonshockable rhythms: Asystole/bradycardia (26)—not separated	No improved survival in paced bradycardia or asystole patients. Only 2 patients survived, and survival could be directly attributed to pacing in only 1 of them. Asystole or bradycardia resistant to pharmacological treatments is a possible indicator of profound myocardial damage that may not benefit from pacing

Madsen et al. [14]	USA, 1987	91	Nonshockable rhythms: Asystole (59) Electromechanical dissociation (27) Pulseless idioventricular rhythm (PIVR) (5)	There was no significant difference in the frequency of electrical capture among the various rhythms (*p*=0.21). One patient survived to be admitted to the hospital but did not survive to be discharged

Rosenthal et al. [15]	Denmark, 1988	24	Nonshockable rhythms: Asystole (2) 3^rd^ degree AV block (9) Sick sinus node syndrome (12) 3rd degree SA block (1)	Supports the feasibility of transcutaneous pacing with no observed side effects. One of the two asystole cases survived

Olson et al. [16]	UK, 1988	32	Nonshockable rhythms: Asystole (21) Bradycardia (11)	Of the 21 patients presenting in asystole, 11 showed possible electrical capture only. Transcutaneous pacing may be beneficial for profound bradycardia however unlikely in asystole cases

Syverud et al. [17]	USA, 1985	12	Nonshockable rhythms: Asystole (5) PIVR (6) Complete heart block (1)	Only 1 successful capture and all patients died in the emergency department. Emphasis on the requirement of early initiation of pacing in future studies

Hedges et al. [18]	USA, 1986	19	Nonshockable rhythms: Asystole (9) PIVR (9) Sinus bradycardia (1)	Separated patients according to time of initiation of pacing. Relatively better results in patients who were paced earlier during bradyasystolic cardiac arrests. Five patients were transcutaneously paced within 5 minutes of cardiac arrest (G1), and 14 were paced between five and 20 min (G2). Two of the G1 patients were admitted and subsequently recovered full neurological and prearrest cardiac function. No patients with full neurologic recovery were in G2 (*p*=0.06)

Paris et al. [19]	USA, 1987	89	Nonshockable rhythms: Asystole & significant bradycardia (not specified)	In paced asystole and hemodynamically unstable bradycardia, pacing did not lead to notable improvements in patient outcomes. In multivariate analysis, presenting an initial rhythm of ventricular fibrillation or tachycardia was correlated to enhanced survival (*p* < 0.05). A short time to PACE was associated with admission to the hospital (*p*=0.20)

Barthell et al. [20]	USA, 1985	112	Nonshockable rhythms: Asystole (55) PIVR (44) Supraventricular bradycardia (8) Complete heart block (5)	Pulses developed following capture in five of 55 patients (9%) in asystole and in four of 44 patients (9%) with pulseless idioventricular rhythms. No patient with complete heart block or other bradycardias developed a pulse. No patients survived to be discharged from the hospital. Poor survival rates were observed despite pacing. Emphasis on the need to initiate pacing shortly after the onset of hemodynamically significant bradycardia or cardiac arrest

Cummins et al. [21]	USA, 1988	103	Nonshockable rhythms: Asystole (62) Electromechanical dissociation (35) Hypotensive bradycardia (6)	No significant difference was observed in survival or resuscitation rates when comparing paced and nonpaced asystole and electromechanical dissociation cases. 22 (21.4%) treated with pacing vs 28 (20.6%) without pacing were alive at arrival to hospital (*p*=0.90) and 7 (6.8%) vs 6 (4.4%) were discharged (*p*=0.71) On the other hand, the bradycardia cases benefitted from external pacing and showed promising results

O'Toole et al. [22]	USA, 1993	112	Nonshockable rhythms: Asystole (112)	No difference in outcome was observed between paced and nonpaced asystole cases. Implementation of out-of-hospital transcutaneous pacing for cardiac arrest asystole cases would not be beneficial. The odds ratios for admission (1.4; 95% CI, 0.62–3.1) and discharge (1.2; 95% CI, 0.2–7.5) were not significantly different from 1.0

**Table 2:** Summary of studies assessing postshock pacing in ICD, S-ICD, and WCD patients.

Authors	Country, year	No. of patients	Studied group	Study conclusions
Budeus et al. [40]		314	PSP analyzed in S-ICD patients undergoing DFT (induced arrhythmias in general anesthesia)	PSP required and appropriately initiated in 25.2% patients following the shock. No episodes of induced arrhythmias or any other harm associated with PSP were detected
Zoll et al. [7]	Italy, 2017	1068	Spontaneous arrhythmias	43.4% of the ICD patients successfully paced following shock; there were no adverse reactions related to pacing
Berger et al. [44]	USA, 2023	313	313 patients dying out of hospital when wearing WCD	Postshock asystole occurred in 205 (65.2%) and postshock bradycardia in 111 (35.5%) patients who died OOH after being shocked by a WCD for VF or VT. Implanted pacemakers may not prevent asystole or bradycardia after a WCD shock
Abbott et al. [42]	Germany, 2023	780	Evaluation of BNP levels pre and post DFT in patients without postshock pacing and with different rates and durations of PSP	Patients without postshock pacing showed the highest BNP during the follow-up. Patients with postshock pacing with 90 bpm and duration of 60 s had the most favorable BNP level trends compared to other patients

*Note:* DFT = defibrillation threshold test.

Abbreviations: BNP = blood natriuretic peptide; S-ICD = subcutaneous ICD; WCD = wearable cardioverter defibrillator.

## Data Availability

The entire deidentified dataset, data dictionary, and analytic code for this investigation are available upon request, from the date of article publication by contacting the corresponding author.
